# Predicting malignant potential of subsolid nodules: can radiomics preempt longitudinal follow up CT?

**DOI:** 10.1186/s40644-019-0223-7

**Published:** 2019-06-10

**Authors:** Subba R. Digumarthy, Atul M. Padole, Shivam Rastogi, Melissa Price, Meghan J. Mooradian, Lecia V. Sequist, Mannudeep K. Kalra

**Affiliations:** 1 Department of Radiology, Massachusetts General Hospital, 55 Fruit Street, Boston, MA 02114 USA; 2 Department of Medicine, Massachusetts General Hospital, 55 Fruit Street, Boston, MA 02114 USA; 3 Department of Radiology, Massachusetts General Hospital, Harvard Medical School, 75 Blossom Court, Suite 236, Boston, MA 02114 USA

**Keywords:** Radiomics, Lung cancer, Subsolid nodules, Benign and malignant lung nodules, Chest CT, Follow up CT

## Abstract

**Background:**

To assess if radiomics can differentiate benign and malignant subsolid lung nodules (SSNs) on baseline or follow up chest CT examinations. If radiomics can differentiate between benign and malignant subsolid lung nodules, the clinical implications are shorter follow up CT imaging and early recognition of lung adenocarcinoma on imaging.

**Materials and methods:**

The IRB approved retrospective study included 36 patients (mean age 69 ± 8 years; 5 males, 31 females) with 108 SSNs (31benign, 77 malignant) who underwent follow up chest CT for evaluation of indeterminate SSN. All SSNs were identified on both baseline and follow up chest CT. DICOM CT images were deidentified and exported into the open access 3D Slicer software (version 4.7) to obtain radiomic features. Logistic regression analyses and receiver operating characteristic (ROC) curves for various quantitative parameters were generated with SPSS statistical software.

**Results:**

Only 2/92 radiomic features (cluster shade and surface volume ratio) enabled differentiation between malignant and benign SSN on baseline chest CT (*P* = 0.01 and 0.03) with moderate accuracy [AUC 0.624 (0.505–0.743)]. On follow-up CT, 52/92 radiomic features were significantly different between benign and malignant SSN (P: 0.04 - < 0.0001) with improved accuracy [AUC: 0.708 (0.605–0.811), *P* = 0.04 - < 0.0001]. Radiomics of benign SSN were stable over time, whereas 63/92 radiomic features of malignant SSNs changed significantly between the baseline and follow up chest CT (P: 0.04 - < 0.0001).

**Conclusions:**

Temporal changes in radiomic features of subsolid lung nodules favor malignant etiology over benign. The change in radiomics features of subsolid lung nodules can allow shorter follow up CT imaging and early recognition of lung adenocarcinoma on imaging. Radiomic features have limited application in differentiating benign and early malignant SSN on baseline chest CT.

## Background

Pulmonary nodules (solid and subsolid) are ubiquitous, and often, indeterminate findings on chest CT. The subsolid nodules (SSN) are further classified as pure ground-glass (PGGN) and part-solid nodules (PSN). While most small nodules are benign, some nodules are either malignant or have the potential to become malignant unsystematically. Given the high morbidity and mortality associated with lung cancer, differentiating benign nodules from those with malignant potential is crucial.

The current standard of care for distinguishing benign and malignant pulmonary nodules has disadvantages. Although malignancy can be determined rapidly through invasive biopsy procedures including open surgery, percutaneous image-guided, and transbronchial biopsy. Nonsurgical procedures are can have complications such as pneumothorax and pulmonary hemorrhage. An open lung biopsy can result in prolonged hospitalization and increased morbidity [1–5]. Non-invasive differentiation involves longitudinal chest CT examinations to establish nodule stability and benignity. This approach entails a protracted path to specific diagnosis, patient anxiety, and healthcare costs, particularly for SSN, which can sometimes represent indolent malignancy, and require an observation period lasting up to 5 years or more. Thus, there is a need for development and validation of an accurate, noninvasive, and rapid method for characterizing SSN.

Radiomics has been proposed as a study of extracting computerized, algorithm-based features to quantify phenotypic characteristics of lesions in medical images (CT, MR, and PET). Prior studies have demonstrated that radiomics can differentiate tumor grade, genetic mutation, hypoxia, and angiogenesis [6–13]. Furthermore, estimation of tumor heterogeneity (distribution of pixel values within the tumor) with radiomics is a marker of tumor aggressiveness, treatment response, and survival in oncologic patients [6–13]. Prior publications have demonstrated a role of radiomics for distinguishing benign and malignant solid nodules in lung cancer screening cohorts [14, 15]. However, there are sparse data on the use of radiomics to predict the malignant potential of SSN (PSN and PGGN) on the baseline and follow up chest CT. We hypothesized that radiomics could characterize SSN on baseline chest CT examinations. Histopathology and serial follow-up chest CT were used as a standard of reference to determine the etiology. We assessed if radiomics can differentiate benign and malignant SSN on initial or follow up chest CT examinations.

## Materials and methods

Our study was compliant with the Health Insurance Portability and Accountability Act (HIPAA). The institutional review board (IRB) approved the retrospective study. We have no financial disclosures relevant to this study.

### Patient and SSN characteristics

The study included 36 adult patients (5 men; 31 women; Table 1) who underwent serial chest CT examinations for evaluation of one or more indeterminate SSN. All patients had resection or biopsy of at least one SSN to determine the final histology. There were 108 SSN in 36 patients included in the study [1 SSN (*n* = 2 patients), 2 SSN (*n* = 11 patients), ≥ 3 SSN (*n* = 23 patients)].

**Table 1:** Patient demographics and SSN characteristics

Number of patients	36
Male: Female	5:31
Number of SSNs (benign: malignant)	108 (31:77)
Patients with ≥3 SSNs	23 (36)
Mean age at baseline	69 ± 8 years
Mean age at follow-up	73 ± 8 years
Mean time intervals between baseline and follow-up	55 ± 32 months
Average size of SSNs at baseline (benign: malignant)	13.2 ± 6 mm: 12.7 ± 7 mm
Average size of SSNs at follow-up (benign: malignant)	13.3 ± 6 mm: 21.3 ± 17 mm

Our study included patients with SSN that did not have specific features favoring malignancy such as prominent solid component (> 5 mm) or spiculations; these patients underwent follow up chest CT to assess stability or change in SSN. Nodules were deemed malignant if there was histologic proof of malignancy, or a greater than or equal to 25% increase in their size, a significant subjective increase in attenuation, or development of a solid component, over follow up chest CT examinations. The benign SSN were confirmed at histology, or from their stability over serial follow-up chest CT examinations. There were 31 benign and 77 malignant SSN (all adenocarcinoma). The patients did not have malignancy other than lung adenocarcinoma and were also negative for interstitial lung disease or infection in the lungs by imaging.

All SSN were assessed twice, at the baseline (mean age 69 ± 8 years) and the final follow-up (mean age 73 ± 8 years) chest CT examinations. Mean time interval between baseline and final follow-up chest CT exams was 55 ± 32 months. The mean sizes of SSN at baseline and final follow-up chest CT were 13.2 ± 6 mm and 13.3 ± 6 mm (benign SSN), and 12.7 ± 7 mm and 21.3 ± 17 mm (malignant SSN) (Table 1).

The included patients were identified from a database maintained by the medical thoracic oncology unit of our institution.

### Scan parameters

All chest CT examinations were performed on 16 or 64-channel multi-detector-row CT scanners (GE Healthcare or Siemens Healthineers). Scan parameters included 100–120 kV, 80–200 mA with automatic exposure control (Auto mA, GE; Care Dose4D, Siemens), 0.4–0.5 s gantry rotation time, and 0.9:1 pitch. Transverse images were reconstructed with 1.25–1.5 mm section thickness with 50% overlap and with an intermediate (such as detail kernel) soft tissue reconstruction kernel. All patients underwent routine chest CT of the chest with IV contrast. No patients were scanned using low dose lung cancer screening or lung nodule follow up protocols. The DICOM CT images were de-identified and exported offline from our PACS archive.

### Radiomics

We used 3D slicer (Version 4.7), an open source software package, to analyze the SSN on the exported DICOM CT images. All SSN were assessed on both the baseline and the final chest CT examinations. After uploading the images into the 3D slicer, each SSN was segmented manually with a paint function on both the baseline and final chest CT images in lung window. We avoided areas of cystic spaces containing air and artifacts related to motion and beam hardening. After segmenting SSN, Radiomics applet was applied for extracting 92 radiomics features for each nodule, including the first-order statistics assess the distribution of CT numbers or voxel values without considering relationship with the neighboring values. These include mean, median, standard deviation, maximum, minimum, entropy (randomness), and skewness and kurtosis of the histogram of values within the region of interest. The second-order or the texture statistics provide a measure of intra-lesion heterogeneity and assess relationships between the voxel values within the region of interest. These include Gray-Level Co-Occurrence Matrix (GLCM, such as homogeneity, dissimilarity, and cluster shade), Gray-Level Run-Length Matrix (GLRLM, such as grey level non-uniformity, run percentage, and run length non-uniformity), Gray Tone Difference Matrix (GTDM, such as coarseness, contrast, complexity and strength) and Grey Level Size Zone Matrix (GLSZM, such as small area emphasis, large area emphasis, and intensity variability). The feature data were exported to Microsoft EXCEL (Microsoft Inc., Redmond, Washington). A single study co-investigator made all measurements in consultation with a fellowship-trained thoracic radiologist (SD, 16 years of experience) to maintain consistency.

The first order statistics rely on the individual pixel values and do not explore their relationships with other pixels. Energy measures the magnitude of voxel values. Large energy indicates the presence of high voxel intensities in the region of interest (ROI). Entropy measures the randomness in the voxel intensities and increases with the number of microstates within the given data. Kurtosis measures the peakedness of the distribution of values; high kurtosis is linked to several outliers, while a low value suggests lack of outliers. In a region with high kurtosis, the pixel values are concentrated around the data outliers instead of their average. Conversely, data with low kurtosis are concentrated around their average. Skewness measures the asymmetry in the distribution of voxel intensities.

GLCM features consider spatial relationships between two pixels. These are estimated from a matrix of single grey values in the ROI, and provide a second-order joint probability function in the given ROI. On the other hand, GLRLM features look, at runs of pixels or a number of pixels of a given grey value in a sequence in a direction (length of a series of pixels with the same gray level value). GLSZM features explore zones of 9-connected pixels with a given grey value and quantify gray level zones as regions of space with connected voxels that share the same gray level intensity [16, 17].

### Statistical analysis

The data were analyzed using SPSS 21 statistical software (IBM, Armonk, NY). Independent two-tailed Student t-tests were used to compare the radiomic features of PSN and PGGN at the baseline and final time points. The *p*-value of 0.05 with a 95% confidence interval was considered significant. Univariate and multivariate logistic regression analyses were performed to determine significant differences between the radiomic features of benign and malignant SSN. Receiver operating characteristic (ROC) curves were generated for various quantitative parameters.

## Results

### Differentiating malignant and benign SSN at baseline CT

Only 2/92 radiomic features (cluster shade and surface volume ratio) were significantly different between malignant and benign SSN at the baseline on the independent sample t-test (*P* = 0.01 and 0.03). The ROC analysis showed that only one radiomic feature (surface-volume ratio, AUC 0.624 (0.505–0.743), *P* = 0.044] was significantly different between the benign and malignant SSN at the baseline chest CT. The surface volume ratio was also the only predictor for differentiating benign and malignant SSN on univariate logistic regression analysis (*P* = 0.006, Nagelkerke R2 = 0.06). The surface volume ratio explained the 6% variance in separating benign and malignant SSN and enabled correct classification of 71.0% of SSN on baseline chest CT.

### Differentiating malignant and benign SSN on follow up CT

**Fig. 1:**
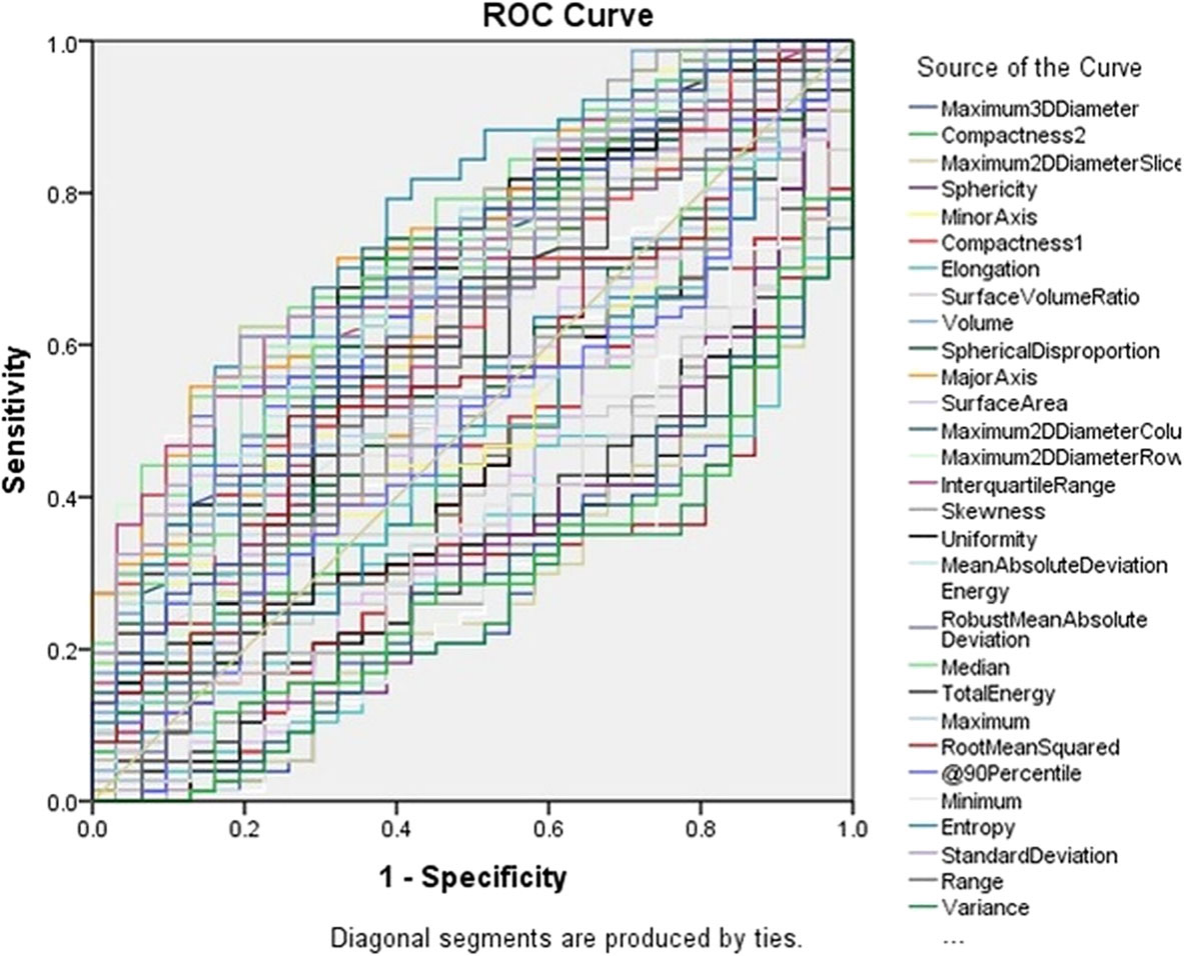
AUC graph of radiomic features on follow-up chest CT for malignant vs. benign SSNs

Fifty-two (52/92) radiomic features (such as entropy, skewness, gray levels, diameter, volume, surface volume, compactness, sphericity, mean HU values and standard deviation) were significantly different for benign and malignant SSN on final follow up CT (P: 0.04 - < 0.0001). ROC analyses demonstrated that 60/92 radiomic features [AUC: 0.708 (0.605–0.811), *P* = 0.04 - < 0.0001] were substantially accurate for differentiating benign and malignant SSN at the final follow up chest CT (Table 2, Fig. 1).

**Table 2:** AUC values for radiomic features on follow-up CT for malignant vs. benign SSN

Test Result Variable	Area	Std. Error	Asymptotic Sig.	Asymptotic 95% Confidence Interval	
				Lower Bound	Upper Bound
Entropy	0.664	0.058	0.008	0.551	0.778
Skewness	0.356	0.057	0.020	0.245	0.467
Compactness	0.389	0.059	0.071	0.273	0.504
Sphericity	0.389	0.059	0.071	0.273	0.504
Mean	0.667	0.054	0.007	0.562	0.773
SD	0.651	0.062	0.014	0.530	0.773
Kurtosis	0.476	0.060	0.701	0.358	0.594
Homogeneity	0.461	0.062	0.530	0.340	0.583
Dissimilarity	0.578	0.064	0.205	0.452	0.704
Cluster Shade	0.357	0.053	0.020	0.252	0.461

The univariate logistic regression analysis showed that entropy was the strongest predictor of benignity and malignancy on follow up CT (*P* = 0.006, Nagelkerke R2 = 0.10). Entropy explained the 10% variance in differentiating benign and malignant lesions on the final follow up CT, and correctly classified 75.0% of benign and malignant PSN and PGGN. Multivariate logistic regression analysis (Backward: Conditional) was also performed with the addition of selective radiomic features (including entropy, kurtosis, skewness, homogeneity, dissimilarity, cluster shade, contrast, gray levels, diameter, volume, surface volume, compactness, sphericity, mean HU values and standard deviation) into the regression model to obtain the final statistics. The logistic regression models were statistically significant (backward: conditional *P* = 0.02 - < 0.0001). The backward conditional model correctly differentiated 82% PSN, and PGGN [sensitivity 90% (CI 81–94%), specificity 61% (CI 42–78%)] as benign or malignant on final follow up CT, slightly increasing the sensitivity compared to the univariate model.

### Changes in benign and malignant SSN over time

**Fig. 2:**
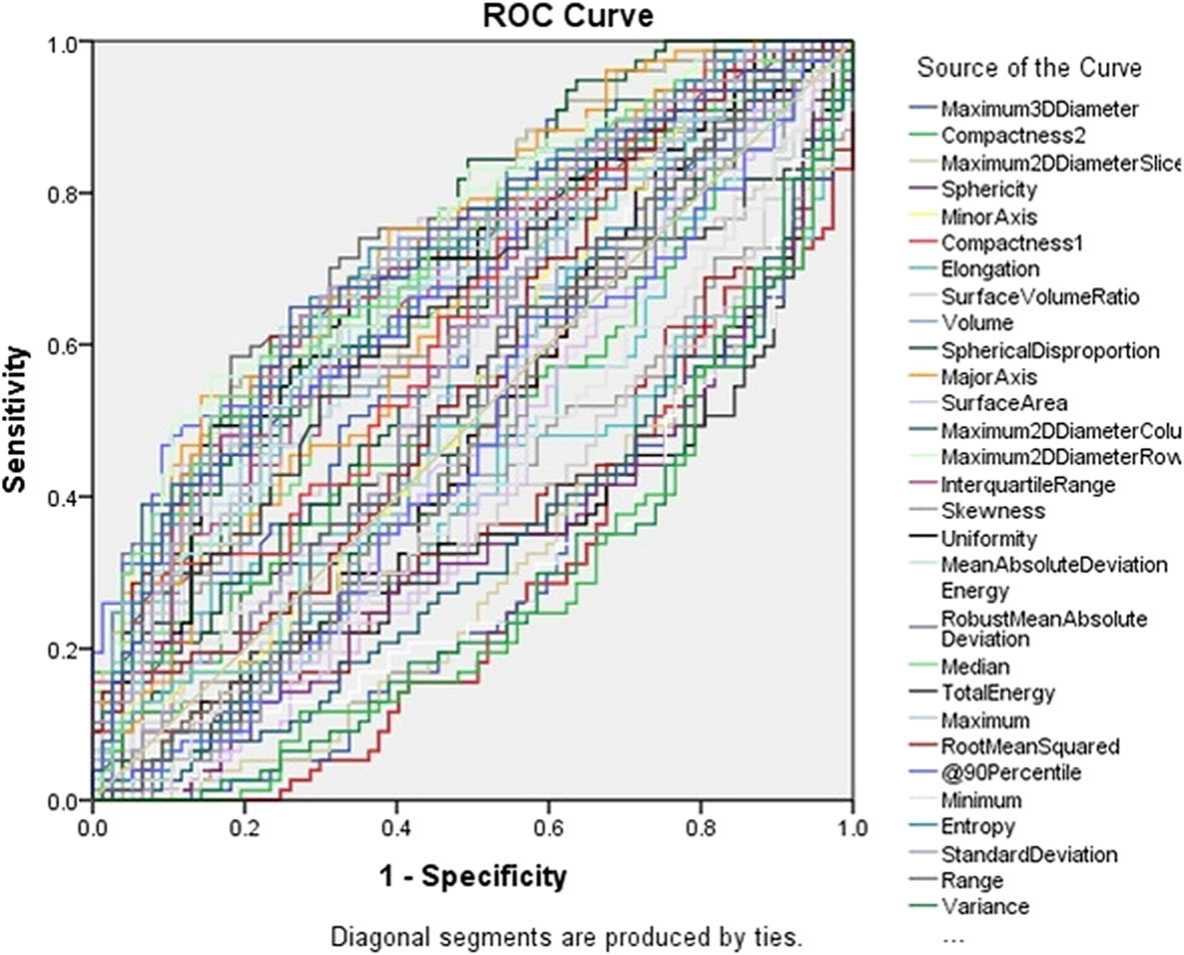
AUC graph of radiomic features for malignant SSNs at baseline CT vs. follow-up CT

**Fig. 3:**
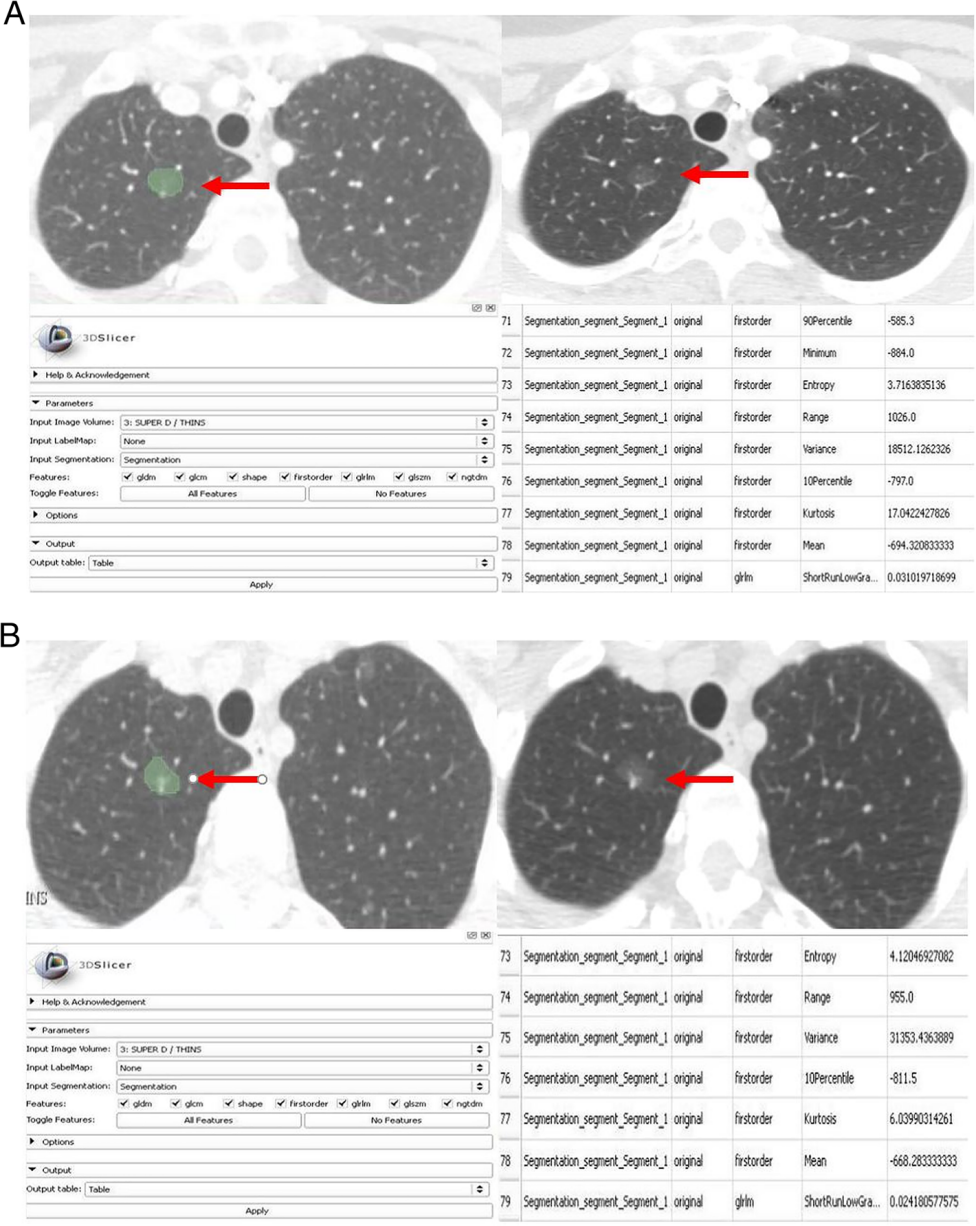
**a** Radiomic analysis of a malignant SSN on baseline chest CT of a 62-year-old woman. **b** Radiomic analysis of a malignant SSN of the same patient 3 years later. Radiomic features (e.g., entropy, kurtosis, mean, grey level variance) were substantially different on the follow-up CT compared to the baseline CT

There were significant statistical differences in 63/92 radiomic features (such as entropy, skewness, gray levels, diameter, volume, surface volume, compactness, sphericity, mean HU values and standard deviation) of malignant SSN on the baseline compared to follow up CT (P: 0.04 - < 0.0001) with a mean area under curve (AUC) of 0.741 (0.664–0.818) (Fig. 2, 3a and b, Table 3). The univariate logistic regression analysis demonstrated that entropy was a significant predictor for differentiating between malignant PSN and PGGN at baseline versus final follow up CT [*P* = 0.01 (1.511–4.475), Nagelkerke R2 = 0.11]. The entropy explained an 11% variance in separating malignant SSN at baseline and final time points and correctly classified/identified 61.0% of malignant SSN at baseline and final time points. The univariate logistic regression for other radiomic features such as kurtosis was not statistically significant: mean (*P* = 0.3).

**Table 3:** AUC values for radiomic features of malignant SSN at baseline CT vs. follow-up CT

Test Result Variable	Area	Std. Error	Asymptotic Sig.	Asymptotic 95% Confidence Interval	
				Lower Bound	Upper Bound
Entropy	0.667	0.044	0.000	0.582	0.753
Skewness	0.410	0.046	0.053	0.320	0.500
Compactness	0.275	0.040	0.000	0.197	0.354
Sphericity	0.275	0.040	0.000	0.197	0.354
Mean	0.642	0.045	0.002	0.555	0.730
SD	0.689	0.043	0.000	0.605	0.773
Kurtosis	0.513	0.047	0.785	0.421	0.605
Homogeneity	0.488	0.047	0.802	0.396	0.580
Dissimilarity	0.570	0.046	0.134	0.479	0.661
Cluster Shade	0.441	0.048	0.205	0.347	0.535

None of the radiomic features were significantly different for benign SSN at baseline versus final time points on the independent sample t-test (*P* = 0.9) and ROC analysis [AUC: 0.566 (0.419–0.714), *P* = 0.4].

## Discussion

Management of SSN is challenging compared to the solid pulmonary nodules of the same size. Several attributes complicate the management of SSN including slow growth, hypometabolism, and nonspecific biopsy findings [18–21]. The current standard of care employ criteria such as increases in size, development of a solid component, or an increase in attenuation on follow up chest CT to predict the malignant potential of SSN. Although most radiomic features (90/92) were also unable to differentiate benign and malignant SSN on baseline chest CT, nodule shape (i.e. surface: volume ratio) and cluster shade (a measure of skewness and uniformity of the GLCM) had a moderate accuracy (AUC 0.505–0.743) for characterizing SSN on baseline chest CT. Conversely, on follow up chest CT, most radiomics (60/92) had higher accuracy (AUC 0.605–0.811) for differentiating benign and malignant SSN. First order statistic, entropy, was the strongest predictor of benign and malignant etiology of the SSN on the follow chest CT. Higher accuracy of radiomics on follow up chest CT compared to the baseline exam was likely due to changes in radiomics of malignant SSN and a lack of change in patients with benign SSN noted in our study.

These results are consistent with other studies, but also differ from other studies on the use of radiomics for characterizing pulmonary nodules into benign and malignant categories [22–27]. Yang et al. have reported that morphologic features such as lesion size, borders, and spiculated margins can help differentiate benign and malignant PGGN [18]. Their study of 1934 subsolid nodules (including 94 benign and 1840 malignant) reported that larger size, well-defined borders, and spiculated margins favor malignant over benign etiology for subsolid nodules [24]. We also found that a shape radiomic feature (surface: volume ratio) helps in the characterization of subsolid nodules on the baseline and follow up chest CT. However, in our study, on baseline CT examinations, other features such as size, attenuation, and borders were unable to distinguish between benign and malignant SSN; these features were only effective on final follow up chest CT. These contradictory observations may be related to different patient and nodule subtypes in our study compared to Yang et al. [24], although it is likely that our study employed quantitative radiomics versus subjective assessment used in the prior study.

Hawkins et al. have reported that radiomics on baseline chest CT have 80 and 79% accuracy in prediction of malignant potential of pulmonary nodules within 1 and 2 years, respectively, using 23 stable features in a random forests classifier [22]. In contradiction, we found that only 2/92 features (cluster shade and surface: volume ratio) enabled distinction between benign and malignant nodules with moderate accuracy. The contradictory observations can be attributed to the fact that our study included PSN and PGGN while Hawkins et al. [22] assessed evaluated different pulmonary nodule subtypes (77% or 338/437 nodules were solid in attenuation in their study).

Yagi et al. evaluated CT radiomic features (such as volume, mass, mean CT value, variance, skewness, kurtosis, entropy, uniformity, and percentile CT numbers) of 115 non-solid nodules (≤ 3 cm diameter) to distinguish adenocarcinoma in situ (AIS), minimally invasive adenocarcinoma (MIA), and invasive adenocarcinoma (IAC) [17]. AIS and MIA had significantly greater skewness, kurtosis, and uniformity values compared with IAC, and CT numbers (90th percentile) and entropy could accurately distinguish AIS-MIA from IAC [17]. Han et al. [25], Chae et al. [26], and Hwang et al. [27] have also assessed PGGN with these histologic subtypes of adenocarcinoma using radiomics. We did not assess these histologic types of adenocarcinoma.

The implication of our study is that most radiomic features cannot differentiate between benign or malignant SSN or predict malignant potential on baseline chest CT. Cluster shade and surface: volume ratio (2/92 radiomics) were significantly different between benign and malignant SSN on baseline chest CT, but their success is limited due to their modest accuracy. On the follow-up chest CT, however, radiomics can accurately differentiate between benign and malignant SSN, particularly to address the most suspicious SSN. This can help abbreviate the duration and frequency of follow up required for an indeterminate subsolid nodule. Currently, based on their size, SSN require to follow up of up to 5 years to rule out malignancy and document stability, as per the recommendations of the Fleischner Society [1].

Our study has limitations. We only assessed the radiomic features on the baseline, and at final follow up chest CT examinations although the change from benignancy to malignancy can occur at any time between the time points. Although it may be valuable to extract radiomics from all intervening chest CT between the baseline and the final follow up exams, this was not possible in our study due to the time-consuming and tedious process of manual image export, uploading, and segmentation of all chest CT examinations. Additionally, we extracted radiomic features from a single slice with SSN compared to the entire volume, as it was challenging to parse and separate the nodule from adjacent and passing blood vessels and other anatomical structures. Though the slice-based approach may have reduced accuracy, it is important to note that prior studies have reported no significant differences in the radiomic features for single slice versus volumetric measurements [28–30]. Our study included only routine chest CT and not low dose CT that is used for lung cancer screening and therefore the results may not be applied to these scans. Finally, patients in our study cohort had multiple pulmonary nodules, and therefore the results may or may not apply to a solitary pulmonary nodule.

## Conclusions

In conclusion, a change in radiomic features over time strongly favors the malignant potential of SSN. With time, radiomic features become accurate in differentiating benign from malignant SSN due to phenotypical changes in nodule morphology. Additionally, clusters or groups of radiomic features have better sensitivity and specificity for distinguishing benign and malignant SSN. Unfortunately, radiomic features have limited application in differentiating benign and malignant SSN on baseline chest CT examinations, when nodules are indeterminate by CT morphological features.

## Data Availability

The datasets used and/or analysed during the current study are available from the corresponding author on reasonable request.

## Abbreviations

AUC: Area Under Curve
GLCM: Grey-Level Co-Occurrence Matrix
GLRLM: Grey-Level Run-Length Matrix
GLSZM: Grey Level Size Zone Matrix
PGGN: Pure Ground-glass Nodule
PSN: Part-solid Nodules
ROC: Receiver Operating Characteristic
ROI: Region of Interest
SSN: Subsolid Nodule
